# PROTOCOL: Effectiveness of interventions for improving livelihood outcomes for people with disabilities in low‐ and middle‐income countries: A systematic review

**DOI:** 10.1002/cl2.1184

**Published:** 2021-07-06

**Authors:** Xanthe Hunt, Ashrita Saran, Lena Morgon Banks, Howard White, Hannah Kuper

**Affiliations:** ^1^ Institute for Life Course Health Research, Department of Global Health Stellenbosch University Cape Town South Africa; ^2^ Campbell Collaboration New Delhi India; ^3^ International Centre for Evidence on Disability London School of Hygiene & Tropical Medicine London UK

## Abstract

The objectives of this review are to answer the following research questions: (1) What is the effect size of the effectiveness of interventions to improve livelihood outcomes for people with disabilities in low‐ and middle‐income countries (LMICs), and what is the quality of the evidence base? (2) What works to improve livelihood outcomes for people with disabilities in LMICs? (3) Which interventions appear most effective for different categories of disability? (4) What are the barriers and facilitators to the improvement of livelihood outcomes to people with disabilities?

## 1 BACKGROUND

### 1.1 The problem, condition or issue

The United Nations Convention on the Rights of Persons with Disabilities (UNCRPD) defines disability as “long‐term physical, mental, intellectual or sensory impairments which, in interaction with various barriers, may hinder [a person's] full and effective participation in society on an equal basis with others” (UN, 2006). More than 1 billion persons in the world have some form of disability (World Health Organization, 2011). This figure corresponds to about 15% of the world's population.

Disability and poverty are strongly linked. On a global level, 80% of people with disabilities live in low‐ and middle‐income countries (LMICs) (World Health Organization, 2011). Within countries, disability disproportionately affects the most disadvantaged sector of the population (Banks, Kuper, et al., 2017). Disability is significantly associated with not only poverty, but also lower educational attainment, lower employment rates, and higher medical expenditures, leading scholars to identify the risk of experiencing “multidimensional poverty” (poverty across multiple domains) as extremely high in this population (Mitra et al., 2013). This relationship—between disability and poverty—is bidirectional, and driven by a number of factors and proposed mechanisms; for instance there are high costs associated with many of types of impairments, and people with disabilities are often excluded from opportunities to learn and earn, and so people with disabilities may “fall into” poverty (Braithwaite & Mont, 2009; Mitra, 2018; Mitra et al., 2011, 2013; Palmer, 2011). Conversely, people who are living in poverty may be more vulnerable to injury and illness, and thus at increased risk of acquiring an impairment and experiencing disability (Groce et al., 2011; Palmer, 2011; Trani & Loeb, 2012).

**Figure 1:**
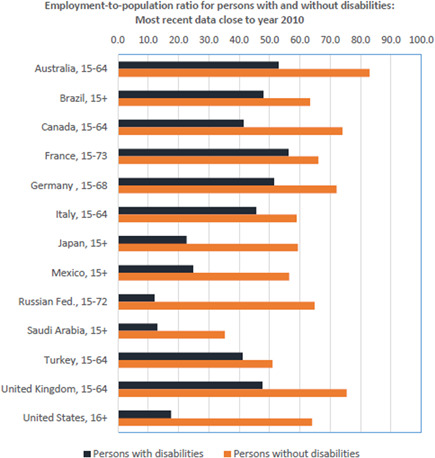
Employment‐to‐population ratio for persons with and without disabilities: Most recent data close to year 2010 (ILO, 2018)

Of relevance to our review is the first of these mechanisms, from disability to poverty. The widespread exclusion of people with disabilities from livelihood opportunities is one of the drivers of the relationship of disability to poverty and is the focus of a substantial literature (Banks & Polack, 2014; World Health Organization, 2011). The 2018 UN Flagship Report on Disability and Development highlighted the large gap in employment between people with and without disabilities (UNDESA, 2018). They reported that across eight geographical regions, the employment to population ratio for people with disabilities aged ≥15 years was 36% compared to 60% for people without disabilities. This employment gap was observed in all regions of the world. The exclusion of people with disabilities from employment is also repeatedly shown in the literature, as illustrated in Figure 1, although these international comparisons must be made with caution due to differences in how disability and employment (especially informal employment) are measured.

There are complexities to the relationship between employment and disability. Disability is not a homogenous category and the experience of exclusion from employment and poverty will vary by gender, impairment type and context. Women already frequently face discrimination in terms of livelihood inclusion, and this may be compounded for women with disabilities (World Health Organization, 2010a). For instance, the World Health Surveys used consistent methods to measure these constructs across 51 countries, and showed that employment levels were lower in men with disabilities (53%) compared to nondisabled men (65%), and also among women with disabilities (20%) compared to nondisabled women (30%) (World Health Organization, 2010a). Exclusion may also vary by impairment type, as people with mental health conditions or intellectual impairments or other “invisible” disabilities (i.e., disabilities which are not readily apparent to others, such as psychosocial disabilities) may be particularly at risk of exclusion from employment (World Health Organization, 2010a), or face resistance when requesting necessary employment accommodations (Prince, 2017). Although data are lacking, people with disabilities may be particularly left behind within humanitarian settings in terms of livelihood inclusion.

Another consideration is that employment alone is not the only pertinent measure of exclusion. Multiple studies have shown that when people with disabilities do work it is more likely to be in the informal sector, part‐time and for lower wages (Banks & Polack, 2014; World Health Organization, 2011). This pattern is illustrated by Figure 1, again with the caveat that differences in measurement of disability and employment (especially informal employment) make international comparisons difficult. The inequity in employment associated with disability occurs despite the fact that almost all jobs can be done by people with disabilities, in particular, if the right supports are in place. However, it is unclear which interventions are most effective at improving employment inclusion and outcomes among people with disabilities in LMICs, and this question has not been previously explored through a systematic review.

It is important to focus beyond waged employment alone, to livelihood more broadly. Livelihood encompasses the means through which individuals or households are able to meet their basic needs. It encompasses people's capabilities (Sen, 1993), assets, income and activities required to secure the necessities of life (Hebinck & Bourdillon, 2001). A livelihood is sustainable when it can cope with, and recover from, stress and shocks, and when it can maintain or enhance its capabilities and assets both now and in the future, while not undermining the natural resource base (Chambers & Conway, 1991). Livelihood, therefore, also includes social protection and financial support, as well as individual's skills to be included in employment.

Social protection includes programmes and policies designed to reduce poverty and vulnerability, for instance, by providing social assistance or by promoting efficient labour markets. Social protection can therefore assure that low‐income and vulnerable populations are able to maintain a basic livelihood, including people with disabilities. Indeed, many countries offer a disability allowance or similar scheme. In Korea, for instance, there is a means‐tested and noncontributory public assistance grant, called the National Basic *Livelihood* Security System (emphasis added) (Jeon et al., 2017). The aim of this grant is to support livelihoods—to mitigate poverty and improve the quality of life and capacity to maintain a minimal standard of living, for the low‐income families and vulnerable groups (including people with disabilities) (Jeon et al., 2017). Social protection interventions need to address the inequalities and the processes of social exclusion that people with disabilities face in attaining a livelihood to have a meaningful impact on their livelihood (de Haan, 2017; Schneider et al., 2016; Stienstra & Lee, 2019). Yet, evidence is lacking on whether social protection or other similar interventions are effective at improving livelihoods for people with disabilities, as most studies have focussed on interventions to improve waged employment alone (Banks, Mearkle, et al., 2017; Cramm & Finkenflugel, 2008).

**Figure 2:**
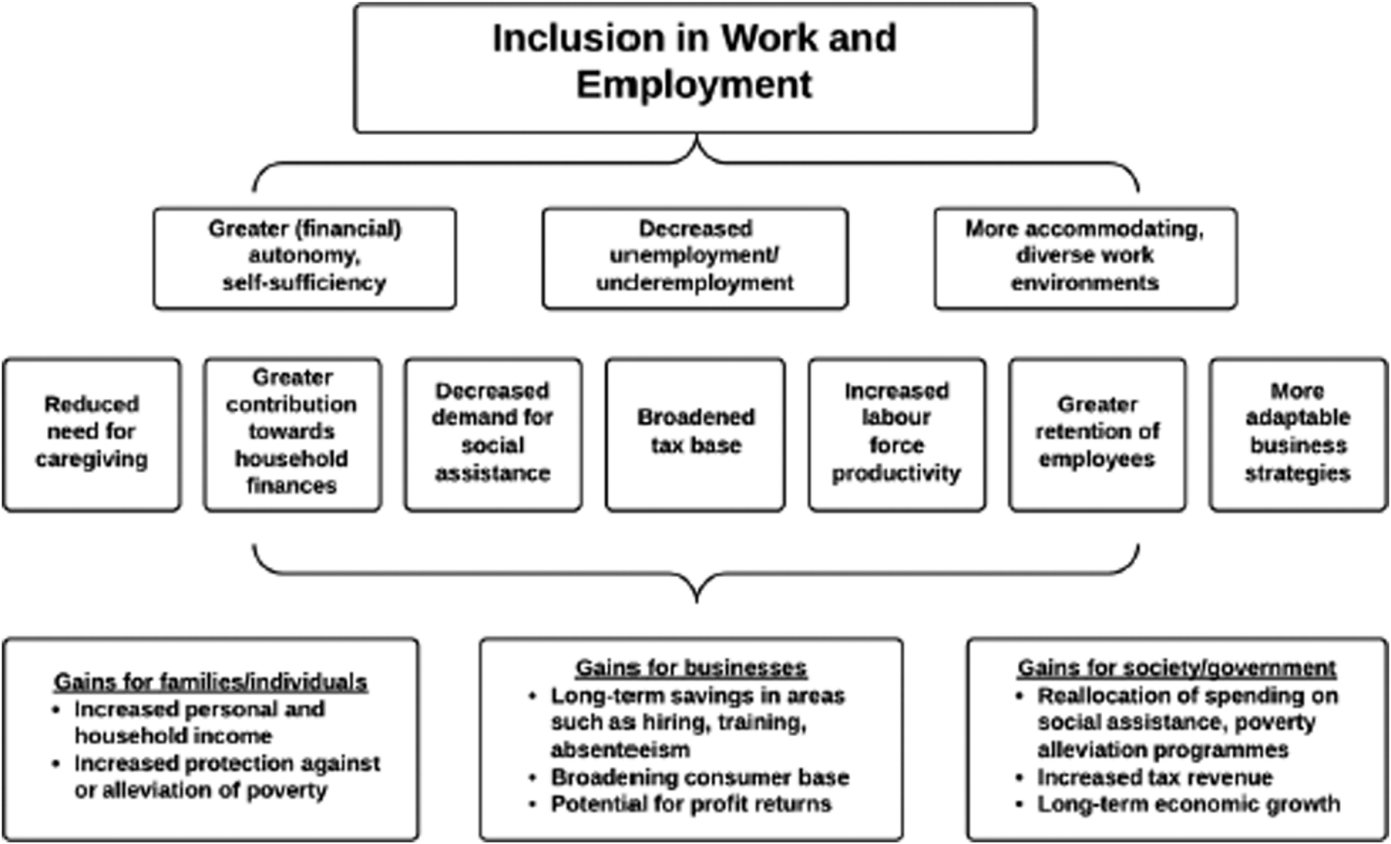
How livelihood can reap gains for people with disabilities (Banks & Polack, 2014)

The financial benefits for people with disabilities of inclusion in livelihood opportunities are obvious (Figure 2) (Banks & Polack, 2014). By definition, improving livelihood outcomes will help people to meet their basic needs. People who are employed will earn income, whether financial or in kind, which will reduce their poverty levels. These benefits will extend beyond the individual to his/her household, as they contribute to the household economy. Financial benefits are also reaped by employers, as they are able to select employees from the full range of skills and abilities, and as evidence suggests that people with disabilities may be particularly loyal and committed employees (UNenable, 2007). Society will also see financial benefits through tax generated from the salary of people with disabilities (Deloitte, 2011). For instance, a report commissioned in 2011 by the Australian Network on Disability showed that closing the gap between labour market participation rates and unemployment rates for people with and without disabilities by one‐third would increase Australia's GDP by $43 billion over the following ten years (Deloitte, 2011).

The nonfinancial benefits of improving livelihood opportunities for people with disabilities must also be emphasised (Figure 2). Employment is a cornerstone of social inclusion, and facilitates friendship and engagement in society. It also promotes human dignity and social cohesion. Fulfilling the right to livelihood inclusion may therefore also help other rights to be met—for instance, the workplace is a key provider of healthcare, and receipt of social protection may help health care and educational costs to be met. These nonfinancial benefits may be particularly pronounced for women, and may include additional gains such as greater protection against abuse, and improved health and educational outcomes of their children.

### 1.2 The intervention

The intervention considered in this review are those that improve livelihood outcomes for people with disabilities. We consider the scope of livelihood in line with the WHO's Community Based Rehabilitation (CBR) Guidelines (World health Organization, 2010b). CBR, which is promoted by the WHO to improve the lives of people with disabilities, has “livelihood” as one of its five pillars (World Health Organization, 2010a). Within the “livelihood” pillar of the CBR matrix, there are five specific components which we use to classify interventions: wage employment, skills development, self‐employment, access to financial services (e.g., micro‐credit schemes, access to bank accounts), and inclusion in social protection programmes. Each of these intervention categories has specific interventions which are named in Table 1 (e.g., vocational training, job placements, and birth registration). Therefore, the CBR will serve as a guiding framework for the intervention categories, as listed below, to realize the full inclusion and empowerment of persons with disabilities. A broad range of capital is needed to improve livelihood outcomes for people with disabilities, including financial capital (e.g., social protection), human capital (e.g., health and education/training), social capital (e.g., support) and physical capital (e.g., accessible buildings) (Hanass‐Hancock & Mitra, 2016). We have added two additional categories to the livelihood pillar, namely Assistive Technologies (ATs) and Rehabilitation, and Policies. We will consider interventions that specifically target people with disabilities, as well as mainstream programmes that are inclusive of people with disabilities.

**Table 1:** Livelihoods interventions and intervention subcategories

Intervention category	Intervention subcategory
Skills development	Training opportunities for employment such as vocational training
	Access to basic educational opportunities
	Social and communications skills training
	Business skills training
Self‐employment	Agricultural or nonagricultural
Waged employment	Apprenticeships
	Job searching services
	Overcome physical and social barriers to the workplace
	Job placement
Financial services	Access to credit
	Savings and loans initiatives
Social protection	Health and social insurance schemes
	Cash transfers, in kind transfers (e.g., food for work programmes)
	Birth registration
	Social assistance intervention
AT and rehabilitation	Rehabilitation
	Assistive technology
Policies	International legislation like universal declaration of human rights
	Employment policies (e.g., antidiscrimination, quotas or accessible buildings)

### 1.3 How the intervention might work

It is important to consider the barriers to livelihood opportunities experienced by people with disabilities, to identify how these may be overcome. People with disabilities are not a homogenous group, and the reasons for exclusion will vary for women and men, in different settings, and for people with different impairment types. Nevertheless, barriers can be broadly categorised as being experienced at the level of the System, the Workplace, the Family or the Person (Wapling, 2016).

*System‐level barriers* include the lack of legislation or policies to support the inclusion of people with disabilities in livelihood opportunities. Even where there are good policies, these may not be implemented due to failure to monitor inclusion or to implement incentives or penalties to promote inclusion. Another important concern is inadequate resource allocation to support inclusion (e.g., lack of funds for access to work schemes). Policies may also be inappropriately formulated so that they penalise people with disabilities who work (e.g., create a benefits trap) or establish over‐protective labour laws that discourage firms from employing disabled people.

*Programme‐level barriers* include lack of reasonable accommodation (including AT) and physical accessibility of the workplace, transport or toilets, or the existence of negative attitudes from employers and coworkers towards people with disabilities. Programmes, such as micro‐credit schemes, may also explicitly exclude people with disabilities (e.g., making people with long‐term health conditions ineligible).

*Individual‐level barriers* include the lower level of training or skills of people with disabilities, following their higher risk of exclusion from education, which may make livelihood opportunities more difficult to obtain. People with disabilities may also experience poor health, and require treatment and rehabilitation, which can make full‐time employment more challenging. Depending on the impairment type, people with disabilities may have difficulties with different skills needed in many work environments, such as concentrating and controlled behaviour, and this may reinforce negative attitudes that people with disabilities are not capable of learning or worth investing in. People with disabilities may experience higher costs of working (e.g., need for accessible transport), which creates a barrier to entry into the labour force. Attitudinal barriers may also be important, for instance if relatives discourage a person with disabilities from working in attempts to be protective or if people with disabilities themselves hold negative attitudes through internalising societal stereotypes.

Approaches to improve livelihood inclusion and outcomes for people with must act by targeting the barriers that they experience. In other words, they must operate at the level of the system (e.g., improving policy and legislation), the programme (e.g., making reasonable accommodations) and/or individual (e.g., providing training in new skills). These interventions should address inclusion in livelihood opportunities in the broadest sense, and not focus only on employment alone.

The World Report on Disability describes different approaches to addressing barriers and thereby enhancing livelihood opportunities.

At the *systems‐level*, most countries have laws and regulations in place protecting people with disabilities from discrimination in employment,^1^ but they should be implemented where they are lacking or improved if they are inadequate. Systems‐level interventions may also include instituting requirement for reasonable accommodation in the workplace, implementation of quotas for employment of people with disabilities, establishment of tax incentives to employers, mainstreaming disability into public employment services, or promotion of affirmative action. A concern is that regulations can act as disincentive to the employment of people with disabilities (e.g., due to expense of providing specialist resources, of strong protection of workers' rights), and this must be avoided.

Examples of *programme level* interventions include supported employment (e.g., specialist job training, social firms), sheltered employment (e.g., employment in segregated facilities), specialist employment agencies for people with disabilities and training of nondisabled staff to produce a more inclusive work environment (e.g., equality/bias training, skills/confidence/communication training).

*Individual‐level* interventions include activities such as vocational rehabilitation programmes, which aim to restore the capabilities of people with disabilities so that they can participate in a competitive labour market, or other forms of skill development. Enrolment of individuals in microfinance schemes and social protection may also help people with disabilities meet their basic needs. However, care must be taken that they do not provide disincentive to work. Efforts to change attitudes are also important, so that people with disabilities are seen as capable of productive work.

### 1.4 Why it is important to do this review

The need to include people with disabilities in employment specifically, and in livelihood opportunities more broadly, is recognised by various international policies and UN directives.

The UNCRPD recognises the rights of people with disabilities to work and employment (article 27), including the “opportunity to gain a living by work freely chosen and accepted in a labour market and work environment that is open, inclusive and accessible to persons with disabilities” (UN, 2006). This article also makes reference to the rights of persons with disabilities to access technical and vocational training, opportunities for self‐employment and entrepreneurship, and a good working environment that provides reasonable accommodation. Article 28 of the UNCRPD asserts the rights of persons with disabilities to accessing social protection programmes and poverty reduction programmes.

The Sustainability Development Goals (SDGs) are also relevant to this issue (UN, 2015). SDG1 is to “End poverty in all its forms everywhere”, and includes a specific target to “Implement nationally appropriate social protection systems and measures for all” (emphasis added). Furthermore, SDG 8 is to “Promote sustained, inclusive and sustainable economic growth, full and productive employment and decent work for all”. This goal is ambitious as “decent work for all”, according to the International Labour Organisation (ILO), means opportunities for work that are productive and deliver a fair income, security in the workplace and social protection for families, better prospects for personal development and social integration, freedom for people to express their concerns, organise and participate in the decisions that affect their lives and equality of opportunity and treatment for all women and men (ILO, 2018). “Sustained” and “sustainable economic growth” places emphasis on long‐term endurance. Finally, “inclusive” requires opportunities for work to be equal for different groups, and SDG8 explicitly states that it is inclusive of people with disabilities.

CBR is promoted by the WHO to improve the lives of people with disabilities, and it has “livelihood” as one of its main pillars (World Health Organization, 2010a). The focus on livelihood includes wage employment, but also includes skills development, self‐employment, access to financial services (e.g., micro‐credit schemes), and inclusion in social protection programmes.

In addition, most countries have policies in place protecting people with disabilities from discrimination in employment specifically. Recent examples include the Law on the Rights of Persons with Disabilities adopted in India in 2016 and Indonesia Law no. 8/2016 on Persons with Disabilities.

It is clear that extensive policies are in place promoting livelihood opportunities for people with disabilities. However, existing research does not provide clear conclusions regarding which interventions are most effective to improve livelihood outcomes for people with disabilities in LMICs; nor whether interventions appear more or less effective for different categories of disability. Furthermore, evidence on which interventions are effective to achieve the specified policies have not been systematically reviewed.

Several relevant systematic reviews and protocols do exist that are relevant to the topic, but none which would address the stated objectives of this review.

Two relevant Campbell reviews have been completed. Iemmi et al sought to assess the effectiveness of CBR for people with disabilities in LMICs, but interventions to improve livelihood outcomes that do not operate through CBR were not be identified for this review (Iemmi et al., 2015). Tripney et al assessed the effectiveness of interventions to improve the labour market situation of adults with physical and/or sensory disabilities in LMICs (Tripney et al., 2015). This review identified 14 eligible studies, which generally found positive impacts of the interventions, despite concerns about the quality of the data. While this latter review is relevant to the current proposed review, it did not include interventions aimed at people with psychosocial disabilities, nor did it address broader livelihood outcomes (e.g., social protection, access to financial services). There is also likely to be relevant papers published since the review was undertaken.

There is a broader existing pool of reviews which focus on specific aspects of the central question of which interventions are effective at improving livelihood outcomes for people with disabilities. These reviews are restricted in terms of:

− **Impairment type/condition included:** Several reviews have been undertaken, or are planned, which focus on livelihood outcomes for people with specific impairments or conditions. Many of these addressed only employment among people with musculoskeletal conditions (Alexander et al., 2017; Sundstrup et al., 2018). Reviews also exist or are planned that focus on other conditions or impairment types, such as people with autism (Westbrook et al., 2013), acquired brain injury (Batavia et al., 2017), stroke (Chan et al., 2013) or mental health conditions (Suijkerbuijk et al., 2017). However, reviews are lacking addressing disability holistically.
− **Eligible livelihood outcomes:** Reviews have been undertaken or are planned that focus only on restricted outcomes related to livelihood. As an example, Gensby et al. (2012) addressed the effectiveness of workplace‐based disability management programs for promoting return‐to‐work outcomes, while Alexander et al. (2017) focussed on work participation. Banks, Mearkle, et al. (2017) considered studies on what is effective to improve inclusion and outcomes for people with disabilities. Here too, data are lacking despite the fact social protection programmes and financial schemes are widely promoted globally in efforts to alleviate poverty.
− **Other socio‐demographic restrictions:** Several reviews exist focussed only on interventions for young adults (Arif, 2018).

Another concern with existing reviews is that many are still at the protocol phase and have not yet been published (e.g., Alexander et al. 2017; Sundstrup et al., 2018). Furthermore, most existing reviews have either identified no eligible studies (e.g., Westbrook et al., 2013), or only studies from high income settings (e.g., Gensby et al., 2012, or Arif, 2018).

There is consequently a need for a review assessing the overall literature on effectiveness of interventions to improve livelihood for people with disabilities, including broad livelihood outcomes, and focussing on LMICs.

## 2 OBJECTIVES

The objectives of this review are to answer the following research questions:

1. What is the effect size of the effectiveness of interventions to improve livelihood outcomes for people with disabilities in LMICs, and what is the quality of the evidence base?
2. What works to improve livelihood outcomes for people with disabilities in LMICs?
3. Which interventions appear most effective for different categories of disability?
4. What are the barriers and facilitators to the improvement of livelihood outcomes to people with disabilities?

## 3 METHODS

### 3.1 Criteria for considering studies for this review

#### 3.1.1 Types of studies

Eligible study designs are defined on the basis of being a type of impact evaluation. Descriptive studies of various designs and methodologies are not included because they, unlike impact evaluations, cannot speak to the question of effect. To answer the question posed by this review “What works to improve livelihood outcomes for people with disabilities in LMICs?”, evidence of effect is required.

Eligible designs include those in which one of the following is true:

a) Participants are randomly assigned (using a process of random allocation, such as a random number generation),
b) A quasi‐random method of assignment has been used,
c) Participants are nonrandomly assigned but matched on pretests and/or relevant demographic characteristics (using observables, or propensity scores) and/or according to a cut‐off on an ordinal or continuous variable (regression discontinuity design),
d) Participants are nonrandomly assigned, but statistical methods have been used to control for differences between groups (e.g., using multiple regression analysis or instrumental variables regression),
e) The design attempts to detect whether the intervention has had an effect significantly greater than any underlying trend over time, using observations at multiple time points before and after the intervention (interrupted time‐series design),
f) Participants receiving an intervention are compared with a similar group from the past who did not (i.e., a historically controlled study), or
g) Observations are made on a group of individuals before and after an intervention, but with no control group (single‐group before‐and‐after study).

#### 3.1.2 Types of participants

The target population are people with disabilities living in LMICs, including people with physical, sensory, intellectual, cognitive and psychosocial (i.e., arising from a mental health condition) impairments. Population subgroups of interest include: women, children (particularly vulnerable children, e.g., those in care), different impairment groups, conflict (conflict and post‐conflict settings), migrants/refugees/internally displaced people, and ethnic minority groups.

#### 3.1.3 Types of interventions

As indicated in SDG guidelines to generate an inclusive and global dialogue, implementing the SDGs must be in line with, and build upon, existing international and national commitments and mechanisms. The WHO recognises CBR as a comprehensive and multi‐sectoral strategy to equalize opportunities and include people with disabilities in all aspects of community life. Therefore, the CBR will serve as a guiding framework for the intervention and outcome categories as listed below to realize the full inclusion and empowerment of persons with disabilities. There are no restrictions on comparators/comparison groups, however a study must have *both* an eligible intervention *and* an eligible outcome to be included. Eligible interventions relate to the livelihood pillar of the CBR matrix. These are:

**Table:** 

Intervention category	Intervention subcategory
Skills development	Training opportunities for employment such as vocational training
	Access to basic educational opportunities
	Social and communications skills training
	Business skills training
Self‐employment	Selling of goods and services by PWDs
Waged employment	Apprenticeships
	Job searching services
	Facilitate physical access to the workplace
	Job placement
Financial services	Access to credit
	Savings and loans initiatives
Social protection	Health and social insurance schemes
	Cash transfers and remittances
	Birth registration
	Social assistance intervention
AT and rehabilitation	Rehabilitation
	Assistive technology
Policies	International legislation like universal declaration of human rights
	Employment policies

#### 3.1.4 Types of outcome measures

Eligible outcomes will relate to the livelihood pillar of the CBR matrix. All outcomes will be relevant regardless of whether they are primary outcomes, or secondary outcomes. It is important to note that if the primary study does not have *both* an eligible intervention *and* an eligible outcome then it will be excluded. The outcomes of interest include those outlined in Table 2.

**Table 2:** Livelihoods outcomes and outcome subcategories

Outcome domain	Outcome subcategory
Acquisition of skills for the workplace	Technical skills
	Business skills
	Social and communication skills
	Basic educational competencies
Access to job market	People with disability are able to engage in job searching
	Physical and social barriers to employment are removed
Employment in formal and informal sector	Entrepreneurship and informal sector participation
	Waged employment and formal sector participation
Income and earnings from work	Men and women with disability have paid and decent work in the formal and informal sector on equal bases with others
	Women and men with disability earn income through their own chosen economic activities
Access to financial services such as grants and loans	Men and women with disability have access to grants, loans and other financial services on an equal basis with others
	Men and women with disability participate in local saving and credit schemes
Access to social protection programs	Men and women with disability access formal and informal social protection measures they need

#### 3.1.5 Duration of follow‐up

Any duration of follow‐up will be included.

#### 3.1.6 Types of settings

All settings will be eligible, provided that the study is situated within a LMIC, as defined by the World Bank (https://datahelpdesk.worldbank.org/knowledgebase/articles/906519-world-bank-country-and-lending-groups).

### 3.2 Search methods for identification of studies

The search for will comprise: (1) an electronic search of databases and sector‐specific websites, and (2) screening of all included studies in the instances where reviews are identified.

#### 3.2.1 Electronic searches

A search of the following electronic databases will be conducted by the authors.

- MEDLINE(R)
- Embase Classic+Embase
- PsycINFO
- CAB Global Health
- CINAHL
- ERIC
- Scopus
- Web of Science (Social Sciences Citation Index)
- WHO Global Health Index

MEDLINE, Embase, PsychINFO and CAB Global Health will be searched through OVID and ERIC and CINAHL through Ebsco. PubMED through NCBI.

Search strategies will be tailored for each of the databases. The main search strategy will be as follows, using English as the search language:

**POPULATION:** (disable* or disabilit* or handicapped) **OR** (physical* or intellectual* or learning or psychiatric* or sensory or motor or neuromotor or cognitive or mental* or developmental or communication or learning) **OR** (cognitive* or learning or mobility or sensory or visual* or vision or sight or hearing or physical* or mental* or intellectual*) adj2 (impair* or disabilit* or disabl* or handicap*) **OR** (communication or language or speech or learning) adj5 (disorder*) **OR** (depression or depressive or anxiety or psychiat* or well‐being or quality of life or self‐esteem or self perception) adj2 (impair* or disabilit* or disabl* or handicap*) **OR** mental health **OR** (schizophreni* or psychos* or psychotic or schizoaffective or schizophreniform or dementia* or alzheimer*) adj2 (impair* or disabilit* or disabl* or handicap*) **OR** (mental* or emotional* or psychiatric or neurologic*) adj2 (disorder* or ill or illness*) **OR** (autis* or dyslexi* or Down* syndrome or mongolism or trisomy 21) **OR** (intellectual* or educational* or mental* or psychological* or developmental) adj5 (impair* or retard* or deficien* or disable* or disabili* or handicap* or ill*) **OR** (hearing or acoustic or ear*) adj5 (loss* or impair* or deficien* or disable* or disabili* or handicap* or deaf*) **OR** (visual* or vision or eye* or ocular) adj5 (loss* or impair* or deficien* or disable* or disabili* or handicap* or blind*) **OR** (cerebral pals* or spina bifida or muscular dystroph* or arthriti* or osteogenesis imperfecta or musculoskeletal abnormalit* or musculo‐skeletal abnormalit* or muscular abnormalit* or skeletal abnormalit* or limb abnormalit* or brain injur* or amput* or clubfoot or polio* or paraplegi* or paralys* or paralyz* or hemiplegi* or stroke* or cerebrovascular accident*) adj2 (impair* or disabilit* or disabl* or handicap*) **OR** (physical* adj5 (impair* or deficien* or disable* or disabili* or handicap*) **OR** people with disabilities/or children with disabilities/or people with mental disabilities/or people with physical disabilities/**OR** abnormalities/or exp congenital abnormalities/or exp deformities/or exp disabilities/or exp malformations/**OR** exp mental disorders/or exp mental health/or learning disabilities/or paralysis/or paraparesis/or paraplegia/or poliomyelitis/or hearing impairment/or deafness/or people with hearing impairment/or vision disorders/or blindness/or people with visual impairment/

**STUDY DESIGN:** (controlled clinical trial/or randomized controlled trial/or equivalence trial/or pragmatic clinical trial/or case‐control studies/or retrospective studies/or cohort studies/or follow‐up studies/or longitudinal studies/or prospective studies/or epidemiologic methods/or epidemiologic studies/or controlled before‐after studies/or cross‐sectional studies/or interrupted time series analysis/or control groups/or cross‐over studies/or double‐blind method/or matched‐pair analysis/or meta‐analysis as topic/or random allocation/or single‐blind method/or "retraction of publication"/or case reports/**OR** (random or placebo or single blind or double blind or triple blind or cohort or ((case or cohort or follow up or follow‐up) adj2 (control or series or report or study or studies)) or retrospective or (observ adj3 (study or studies)))

**LOCATION:** Developing Countries **OR** Africa/or Asia/or Caribbean/or West Indies/or Middle East/or South America/or Latin America/or Central America/**OR** (Africa or Asia or Caribbean or West Indies or Middle East or South America or Latin America or Central America) **OR** ((developing or less* developed or under developed or underdeveloped or middle income or low* income or underserved or under served or deprived or poor*) adj (countr* or nation? or population? or world or state*)) **OR** ((developing or less* developed or under developed or underdeveloped or middle income or low* income) adj (economy or economies)) **OR** (low* adj (gdp or gnp or gross domestic or gross national)) **OR** (low adj3 middle adj3 countr*) **OR** (lmic or lmics or third world or lami countr*) **OR** transitional countr*

#### 3.2.2 Searching other resources

We will search the reference lists of identified recent papers and reviews. To ensure maximum coverage of unpublished literature, and reduce the potential for publication bias, we will search the following organisational websites and databases using the keyword search for unpublished grey:

- ILO
- DFID (including Research for Development [R4D])
- UNESCO
- WHO
- Disability Programme of the United Nations Economic and Social Commission for Asia and the Pacific
- United States Agency for International Development

Dissertation Abstracts, Conference Proceedings and Open Grey.

- Humanity and Inclusion (HI) http://www.hi-us.org/publications
- CBM https://www.cbm.org/Publications-252011.php
- Plan international https://plan-international.org/publications

### 3.3 Data collection and analysis

A key framing note for the present section is that we do not anticipate conducting a meta‐analysis. If we encounter a cluster of studies which could be analysed we will develop a coding and analysis sheet for this but based on the rapid evidence assessment which preceded this SR, this is deemed unlikely.

#### 3.3.1 Description of methods used in primary research

We will use EppiReviewer (https://eppi.ioe.ac.uk/) to help assess the search results. EppiReviewer is a web‐based software program for managing and analysing data for literature reviews and has been developed for all types of systematic review such as meta‐analysis, framework synthesis and thematic synthesis. In our review, EppiReviewer will be used for bibliographic management, screening, coding and data synthesis.

Unique references will be screened for relevance by title and abstract by two independent reviewers with disagreement resolved by third reviewer. The full text of potentially relevant articles will also be screened independently by two independent reviewers with disagreement resolved by third reviewer for inclusion. Any discrepancy will be resolved by consensus and discussion with the senior author (H. K.). The screening checklist will also be reviewed by H. K. and H. W.

Eligibility will be assessed using a predesigned form based on the inclusion criteria. Any and all changes to these criteria will be reported in the final SR. Articles excluded at this stage will be reported in a table with reasons for exclusion. We will report interrater reliability for study identification.

The screening process will be reported using a PRISMA flow chart.

The screening checklist (Annex 1) will include the following:

1. Does the study include a relevant intervention AND a relevant outcome?
  a) Skills development
  b) Self‐employment
  c) Waged employment
  d) Financial services
  e) Social protection
  f) Employment in formal and informal sector
  g) Access to job market
  h) Control over own money
  i) Access to financial services such as grants and loans
  j) Poverty and out‐of‐pocket payment
  k) Access to social protection programs
  l) Participation in development of inclusive policies
2. Is the study conducted with people with disabilities living in LMICs?
3. Is the study one in which participants are randomly assigned or quasi‐randomly assigned, or where nonrandom assignment has been done, but participants have been matched on pretests and/or relevant demographic characteristics or statistical methods have been used to control for differences between groups; or where the design attempts to detect whether the intervention has had an effect significantly greater than any underlying trend over time, using observations at multiple time points before and after the intervention (interrupted time‐series design); or where participants receiving an intervention are compared with a similar group from the past who did not (i.e., a historically controlled study); or where observations are made on a group of individuals before and after an intervention, but with no control group (single‐group before‐and‐after study).

#### 3.3.2 Criteria for determination of independent findings

Multiple publications of the same study will be examined as a single study.

#### 3.3.3 Selection of studies

Screening will be a two‐stage process of first screening by title and abstract and then full text. Screening will be undertaken independently by two screens, with a third‐party arbiter in case of disagreement.

#### 3.3.4 Data extraction and management

Two review authors (A. S. and X. H.) will independently code and extract data from included studies. A coding sheet will be piloted on several studies and revised as necessary (see Annex 2: Coding sheet). Disagreements will be resolved by consulting a third review author with extensive content and methods expertise (H. W. and H. K.), and will be reported. Data and information will be extracted on: available characteristics of participants, intervention characteristics and control conditions, research design, sample size, risk of bias and outcomes, and results. Extracted data will be stored electronically. Studies will be coded by intervention, outcomes and a range of filters such as study design and location. The coding sheet for this review is included as Annex 2.

The primary studies included in the systematic reviews will also be assessed for eligibility. As such, the systematic review does not include summarised findings of the systematic reviews to avoid duplication.

This evidence assessment is based on studies reporting interventions and outcomes in the domain livelihood. The list of studies coded as such will be screened for eligibility by Ashrita Saran.

#### 3.3.5 Quality assessment and assessment of risk of bias in included studies

Table 3 presents the tool which will be used to assess confidence in study findings. This tool^2^ contains six criteria:

1. Study design (potential confounders taken into account): impact evaluations need either a well‐designed control group, preferably based on random assignment, or an estimation technique which controls for confounding and the associated possibility of selection bias.
2. Masking (RCTs only, also known as blinding): masking helps limit the biases which can occur if study participants, data collectors or data analysts are aware of the assignment condition of individual participants.
3. Presence of a power calculation: many studies may be underpowered, but it is difficult to assess without the inclusion in the study of a power calculation.
4. Attrition can be a major source of bias in studies, especially if these is differential attrition between the treatment and comparison group so that the two may no longer be balanced in preintervention characteristics. The US Institute of Education Sciences What Works Clearing House has developed standards for acceptable levels of attrition, in aggregate and the differential, which we will apply.^3^
5. Clear definition of disability: for a study to be useful the study population must be clear, which means that the type and severity of disability should be clearly defined, preferably with reference to a widely‐used international standard
6. Clear definition of outcome measures is needed to aid interpretation and reliability of findings and comparability with other studies. Studies should clearly state the outcomes being used with a definition and the basis on which they are measured, preferably with reference to a widely‐used international standard.
7. Baseline balance shows that the treatment and comparison groups are the same at baseline. Lack of balance can bias the results.

**Table 3:** Study quality assessment criteria

	Criterion	Low	Medium	High
1	Study design (Potential confounders taken into account)	Before versus after. Naïve matching	IV, RDD, PSM, double difference	RCT, natural experiment
2	Blinding (RCTs only)	No mention of blinding	Blinding for analysis.	Blinding of data collection (where feasible). Blinding for analysis
3	Losses to follow up are presented and acceptable	Attrition not reported, OR falls well outside WWC acceptable combined levels*	Overall and differential attrition close to WWC combined levels*	Overall and differential attrition within WWC combined levels*
4	Disability/impairment measure is clearly defined and reliable	No definition OR overall attrition >50%	Unclear definition OR Single question item only (e.g., are you disabled)	Clear definition, for example, Washington Group questions, detailed measure of impairment
5	Outcome measures are clearly defined and reliable	No definition	Unclear definition	Clear definition using existing measure where possible
6	Baseline balance (N.A. for before vs. after)	No baseline balance test (except RCT) OR reported and significant differences on more than five measures. PSM without establishing common support	Baseline balance test, imbalance on 5 or fewer measures	RCT, RDD
	Overall confidence in study findings	Low on any item	Medium or high confidence on all items	RCT with high confidence on all items

Confidence in study findings will be rated high, medium or low, for each of the criteria, applying the standards as shown in Table 3. Overall study quality will be the lowest rating achieved across the criteria—the weakest link in the chain principle.

Where a study reports outcomes at more than one point in time it is possible that the study quality varies between those two points for two of the criteria: (1) an RCT may no longer be so if it used a waitlist or pipeline design so the control group has received the treatment (item 1), (2) there may be greater attrition rates at the later point in time. Hence in applying the tool an assessment is made for the earliest and latest outcome measures for items 1 and 4, and overall study quality assessed separately for the two points in time.

##### An example of applying the tool

Table 4 shows the application of the quality assessment tool to the study of Grider (2014) (Grider & Wydick, 2016). This study is a controlled before‐and‐after study, comparing the change in measures of employment (e.g., hours worked per day, income) after receipt of wheelchair in comparison to matched controls using Propensity Score Mapping analyses. As summarised in the table below, many of the study characteristics were appropriate (e.g., large size). However, confidence in the study results was judged to be “medium, because the study did not use a randomised controlled design.

**Table 4:** Application of study quality assessment tool to a sample study

No.	Item		Notes
1	Study design, sampling method is appropriate to the study question	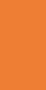	Propensity Score Mapping
2	Adequate sample size, for example, sample size calculations undertaken	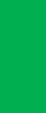	Sample size was not small (120 current wheelchair users and 141 nonwheelchair users), but no power calculation was presented.
3	Attrition	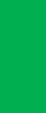	32% of people in the baseline survey were not included in the follow‐up
4	Disability/impairment measure is clearly defined and reliable	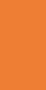	People were classified on the basis of needing a wheelchair, but there was a lack of information on impairment type.
5	Outcome measures are clearly defined and relatable	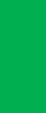	Clear definition of outcomes was used (i.e., hours worked per day, income).
6	Baseline balance	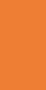	Propensity Score Mapping was used to adjust for baseline differences, although baseline balance was not demonstrated.
	Overall confidence in study findings	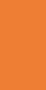	Low on any item

Scoring: Green, “high”; Amber, “medium”; Red, “low”.

#### 3.3.6 Measures of treatment effect

We will collect effect sizes and conduct effect size calculations where none are published, but, as noted, we do not expect that it will be possible to conduct a meta‐analysis, given the diversity of designs, methodologies, measures and rigour across studies in this area.

We will convert these effect sizes to a common metric and will present these in forest plots.

− For continuous outcomes, effects sizes with 95% confidence intervals (CIs) will be calculated, where means and standard deviations are available. If means and standard deviations are not available, we will calculate standardised mean differences (SMDs) from *F* ratios, *t* values, *χ* ^2^ values and correlation coefficients, where available, using the methods suggested by Lipsey and Wilson (2001).
− For dichotomous outcomes, we will calculate odds ratios with 95% CIs. employment outcomes (e.g., presence or absence of gaining competitive employment), are examples of relevant dichotomous outcomes in this review.

There are statistical approaches available to re‐express dichotomous and continuous data to be pooled together (Sánchez‐Meca et al., 2003). To calculate common metric odds ratios will be converted to SMD effect sizes using the Cox transformation. We will only transform dichotomous effect sizes to SMD if appropriate.

When effect sizes cannot be pooled, study‐level effects will be reported in as much detail as possible. Software for storing data and statistical analyses will be RevMan 5.0, Excel, R and Stata 10.0.

#### 3.3.7 Unit of analysis issues

The unit of analysis of interest to the present study are individual people with disabilities, their caregivers, carers, or those working with them. If a study is included with more than two intervention arms, include in the review only intervention and control groups that meet the eligibility criteria. If multiarm studies are included, we will ensure that we do not double‐ count participants, but also ensure that we adequately account for the eligible interventions and their respective effects.

#### 3.3.8 Dealing with missing data

In case of missing information, the author(s) of the original study will be contacted. We will document correspondence with study authors.

#### 3.3.9 Assessment of heterogeneity

We expect great clinical and methodological heterogeneity, as well as some statistical heterogeneity, as the interventions and outcomes of interest are diverse, and outcome measurement highly variable in terms of construct and measurement chosen. Nonetheless, we will code effect sizes. However, if there is too much heterogeneity in the reporting of quantitative data, and the effect sizes, we will synthesise the data only narratively, and without a meta‐analysis.

#### 3.3.10 Assessment of reporting biases

Assessment of reporting biases is covered under the section above “Quality assessment and assessment of risk of bias in included studies”.

#### 3.3.11 Data synthesis

Coding will include: (1) basic study characteristics, (2) narrative summary (including annotation of any adverse effects), (3) summary of findings/results table, and (4) quality assessment. This coding will be conducted by pairs of coders, with comparison and discussion to resolve any discrepancies which arise.

Data will be extracted from the studies according to an extraction table which includes the following sections:

- Target
- Number of individual studies included
- Impairment type
- Outcomes
- Evidence of impact
- Study quality
- Gender analyses conducted
- Humanitarian setting
- Cost‐effectiveness analysis
- Areas of strong evidence
- Level of evidence

We will code effect sizes. We will examine heterogeneity both in the subject matter of included studies (context, intervention and outcomes) and in the reported effect sizes (visually and using I‐squared) (Higgins et al., 2020). If meta‐analysis is appropriate, we will calculate an inverse variance weighted average effect size using a random effects model. However, if there is too much heterogeneity in the reporting of quantitative data, and the effect sizes, we will synthesise the data only narratively, and without a meta‐analysis. Heterogeneity will be assessed by comparing study characteristics such as type of intervention and control comparators, participant demographics, quality of trials (randomisation, blinding, losses to follow‐up) and outcomes measured. Statistical heterogeneity will be assessed visually and by examining the *I* ^2^ statistic, which describes the approximate proportion of variation that is due to heterogeneity rather than sampling error. This will be supplemented by the *χ* ^2^ test, where a *p* < .05 indicates heterogeneity of intervention effects. In addition, we will estimate and present *τ* ^2^, along with its CIs, as an estimate of the magnitude of variation between studies. This will provide an estimate of the amount of between‐study variation. Sensitivity and subgroup analyses will also be used to investigate possible sources of heterogeneity.

The findings will be grouped by suboutcomes, that is: acquisition of skills for the workplace; access to job market; employment in formal and informal sector; income and earnings from work; control over own money; poverty and out‐of‐pocket payment; participation in development of inclusive policies; access to financial services such as grants and loans and access to social protection programs.

For each suboutcome, a narrative summary will be prepared for the main themes and findings, including consideration of where there is strong evidence for effect, where there are evidence gaps, and the quality of the evidence. We will conduct a meta‐analysis of results by subgroup if there are sufficient number of studies (*n* = 4, (Fu et al., 2011) and the level of heterogeneity is not too high.

#### 3.3.12 Subgroup analysis and investigation of heterogeneity

We have not planned subgroup analyses as part of a meta‐analysis given the expected high level of heterogeneity in reporting and effect sizes. However, as noted, we are interested in certain specific populations of people with disabilities, including women, children (particularly vulnerable children, e.g., those in care), different impairment groups, conflict (conflict and post‐conflict settings), migrants/refugees/internally displaced people, and ethnic minority groups. For papers addressing these issues, we will extract effect sizes and if data allows, disaggregate outcome findings by group. However, our expectation is that we will instead be able to provide a narrative description of any apparent notable characteristics of papers addressing these groups, but these findings will be descriptive and tentative.

#### 3.3.13 Treatment of qualitative research

We do not plan to include qualitative research.

## EXTERNAL SOURCES

This systematic review is supported by the UK Department of International Development (DFID) under its support for the Centre for Excellence for Development Impact and Learning (CEDIL) and the Programme for Evidence to iNform Disability Action (PENDA).

## Footnotes

1 Recent examples include the Law on the Rights of Persons with Disabilities adopted in India in 2016 and Indonesia Law no. 8/2016 on Persons with Disabilities.

2 Thanks also to Hugh Waddington (3ie and Campbell IDCG) for suggestions used in developing the tool.

3 See tab. 1, https://homvee.acf.hhs.gov/HomVEE-Attrition-White_Paper-7-2015.pdf.
